# Differential and cooperative effects of IL-25 and IL-33 on T helper cells contribute to cryptococcal virulence and brain infection

**DOI:** 10.1038/s41598-023-37158-1

**Published:** 2023-06-19

**Authors:** Adithap Hansakon, Siranart Jeerawattanawart, Pornpimon Angkasekwinai

**Affiliations:** 1 Department of Medical Technology, Faculty of Allied Health Sciences, Thammasat University, Pathum Thani, 12120 Thailand; 2 Chulabhorn International College of Medicine, Thammasat University, Pathum Thani, 12120 Thailand; 3 Graduate Program in Biomedical Science, Faculty of Allied Health Sciences, Thammasat University, Pathum Thani, 12120 Thailand; 4 Research Unit in Molecular Pathogenesis and Immunology of Infectious Diseases, Thammasat University, Pathum Thani, 12120 Thailand

**Keywords:** Immunology, Microbiology, Diseases, Pathogenesis

## Abstract

The epithelial cell-derived cytokines IL-33 and IL-25 are important mediators in driving type-2 inflammation during *C. neoformans* infection. Nevertheless, the impact of these cytokines in regulating host T helper cell response during *C. neoformans* infection is still unclear. We observed that *C. neoformans* infection promoted a predominant increase of T helper cells that co-expressed IL-25 and IL-33 receptors within the lung during the late infection phase. A comparative transcriptomic analysis of effector T helper cells co-treated with IL-25 and IL-33 revealed a cooperative effect of these cytokines in promoting IL-13 gene expression. Without IL-25 receptor signaling, IL-33 treatment upregulated Th1-associated genes and genes associated with nucleotide metabolism. By contrast, IL-25 had a unique effect in enhancing type-2 cytokines IL-5 and IL-9 and chemokine CCL24, as well as genes in the pathways that are associated with L-arginine metabolisms. Interestingly, this pathogenic T helper cell population that expressed IL-25 and IL-33 receptors was greatly enriched in mice that were infected with high cryptococcal virulence and associated with fungal burdens in the brain. Therefore, our data further provide the additional function of IL-25 and IL-33 in potentiating cryptococcal brain dissemination.

## Introduction

In the past decades, cryptococcal meningitis/meningoencephalitis caused by *C. neoformans* infection has been recognized as a leading cause of death in patients living with HIV/AIDS. Worldwide, more than 180,000 cases of cryptococcal meningitis are reported annually^1–3^. *C. neoformans*-infected patients usually present with mild symptoms or are asymptomatic after inhalation of a basidiospore or desiccated yeast cells from the environment^4,5^. However, without robust immunity, the inhaled *C. neoformans* can be reactivated to grow and proliferate within the lung and cause pulmonary cryptococcosis and/or meningoencephalitis when disseminated to the brain^5,6^. The Th1-type immune response, which enhances pro-inflammatory cytokines, such as IFN-γ, and activates classically activated macrophages (M1) with high fungicidal activity, is crucial in limiting cryptococcal infection^7–9^. Nevertheless, the highly virulent strains of *C. neoformans*, such as H99, can suppress the host immunity by skewing the immune response toward Th2-type immune response^10^. These permissive Th2-type environments promote cryptococcal growth, proliferation, and dissemination through the development of alternatively activated macrophages (M2) influenced by their produced cytokine (e.g., IL-4, IL-5, and IL-13)^10–12^.

The mucosal epithelial cells have been directly implicated in the regulation of Th2-type immune responses by secreting cytokines thymic stromal lymphopoietin (TSLP), IL-33, and IL-17E (IL-25) to initiate type-2 immune response^13^. These cytokines function as alarmins in response to environmental stimuli and tissue damage^14,15^ and drive type-2 inflammation by promoting the production of type-2 cytokines from innate lymphoid cell type-2 (ILC2) and adaptive Th2 cells^15–18^. Previous studies have indicated that infection with *C. neoformans* stimulated lung epithelial cells to secrete IL-25 and IL-33 but not TSLP^19–22^. A mouse model of pulmonary cryptococcosis with *C. neoformans* infection showed an increased expression of receptors for IL-33, ST2, which was correlated with Th2 cell activation^22^. ST2 knockout mice infected with *C. neoformans* had improved survival with reduced lung and brain fungal burden and type-2 immune response^19,21^. In a recent study, an epithelial cell-derived cytokine IL-25 was induced in response to high-virulence but not to low-virulence *C. neoformans* infection^20^. In the mouse model of pulmonary *C. neoformans* infection, the IL-17RB knockout mice that lacked endogenous IL-25-mediated signaling had better survival with reduced fungal burden in the brain but not in the lung^20^. The pulmonary expression of IL-25 was found to intensify the Th2 immune response and prompted disseminated cryptococcal disease development^20^. Although IL-25 was found to exacerbate the pathogenesis of cryptococcosis, how IL-25 participates with IL-33 in regulating Th2-type immune responses during *C. neoformans* infections remains unclear.

In this study, we dissect the cellular and molecular mechanisms that IL-25 coordinates with IL-33 in regulating CD4^+^ T cell responses to contribute to cryptococcal disease pathogenesis. The pulmonary CD4^+^ T cells of *C. neoformans*-infected mice showed a predominant co-expression of IL-17RB and ST2, particularly at the late infection stage. Ex vivo treatment of pulmonary effector T helper cells derived from infected mice with IL-25 and IL-33 synergistically enhanced Th2-type cytokines production. Transcriptome profiles of IL-25- and IL-33-treated pulmonary effector CD4^+^ T cells were analyzed by comparing upregulated genes in *Il17rb*^−/−^ mice and wild-type mice during infection. During *C. neoformans* infection, IL-33 largely contributed to T cell activation, whereas IL-25 signaling was preferentially associated with cell survival, proliferation, and type-2 cytokine/chemokine production. The CD4^+^ T cells that co-expressed IL-17RB and ST2 were positively correlated with cryptococcal brain dissemination. Thus, our data indicate that epithelial cell-derived IL-25 and IL-33 had a differential and combinatorial effect on T helper cells to promote Th2-type inflammation and cryptococcal dissemination.

## Results

### IL-17RB^+^ ST2^+^ T helper cell population is predominately induced within the lung during the late phase of ***C. neoformans*** infection

**Figure 1:**
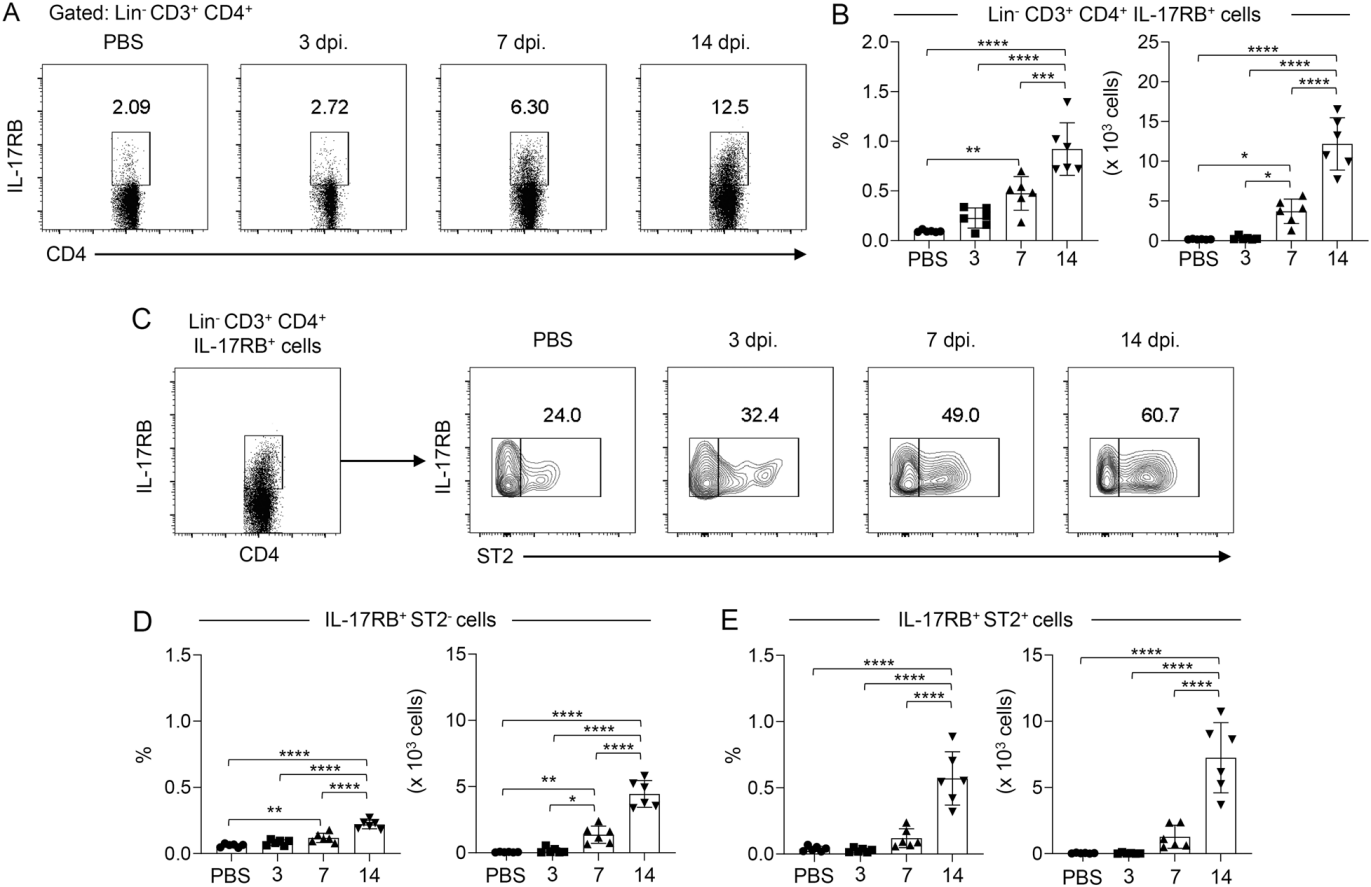
IL-17RB/ST2-expressing CD4^+^ T helper cells are predominantly induced within the lung during the late phase of *C. neoformans* infection. (**A**–**E**) BALB/c mice were treated with PBS or intranasally infected with *C. neoformans* (H99) at 5 × 10^4^ yeast cells/mouse. Pulmonary leukocytes were isolated at 3, 7, and 14 days post-infection and stained with fluorescence-conjugated Ab to lineage markers (Lin; CD11b, CD11c, B220, CD19, DX5, Gr.1, and CD8), CD3, CD4, IL-17RB, and ST2. The population of T helper cells within the lung was analyzed for the expression of IL-17RB and ST2 by flow cytometry. (**A**, **B**) Representative plots (**A**) and frequencies depicted as the percentages and absolute count (**B**) of the total IL-17RB-expressing T helper cell population (Lin^-^ CD3^+^ CD4^+^ IL-17RB^+^). (**C**–**E**) Phenotypic analysis of IL-17RB-expressing T helper cell for the expression of ST2. The gated (**C**) and frequencies of the IL-17RB^+^ ST2^-^ (**D**) and IL-17RB^+^ ST2^+^ (**E**) T helper cells are depicted as the percentages and absolute count. Graphs show individual mice and mean ± SD are pooled from two independent experiments (n = 6 mice per group). Significance was determined using one-way ANOVA with Tukey post hoc analysis (*p < 0.05, **p < 0.01, ***p < 0.001, ****p < 0.0001).

Since the epithelial cell-derived cytokine IL-25 and IL-33 were known to mediate the progression of cryptococcal diseases caused by *C. neoformans* infection by amplifying Th2-type immune response^19–22^, we further analyzed how IL-25 acted together with IL-33 to regulate CD4^+^ T helper cell during infection. Using multicolor flow cytometric analysis, we first characterized the kinetics of IL-17RB, the cognate receptor for IL-25 expression on lung CD4^+^ T helper cells during pulmonary *C. neoformans* H99 infection for 3, 7, and 14 days. Compared with phosphate-buffered saline (PBS)-treated mice, IL-17RB^+^ CD4^+^ T cells were gradually increased within the lung during *C. neoformans* H99 infection and significantly induced at 7 and 14 days post-infection (Fig. 1A,B). Indeed, the IL-33 receptor, ST2, was also induced and co-expressed with IL-17RB in the CD4^+^ T cell population (Fig. 1C). Interestingly, the majority of CD4^+^ T cell significantly induced and enriched at 14 days post-infection was IL-17RB^+^ ST2^+^ cells (Fig. 1C–E). Thus, our data suggested the potential collaboration of IL-25 and IL-33 in regulating adaptive T helper cell responses during *C. neoformans* infection, especially during the late phase of infection.

### IL-25 and IL-33 cooperatively promote the production of cryptococcal-specific type-2 cytokines by T helper cells

**Figure 2:**
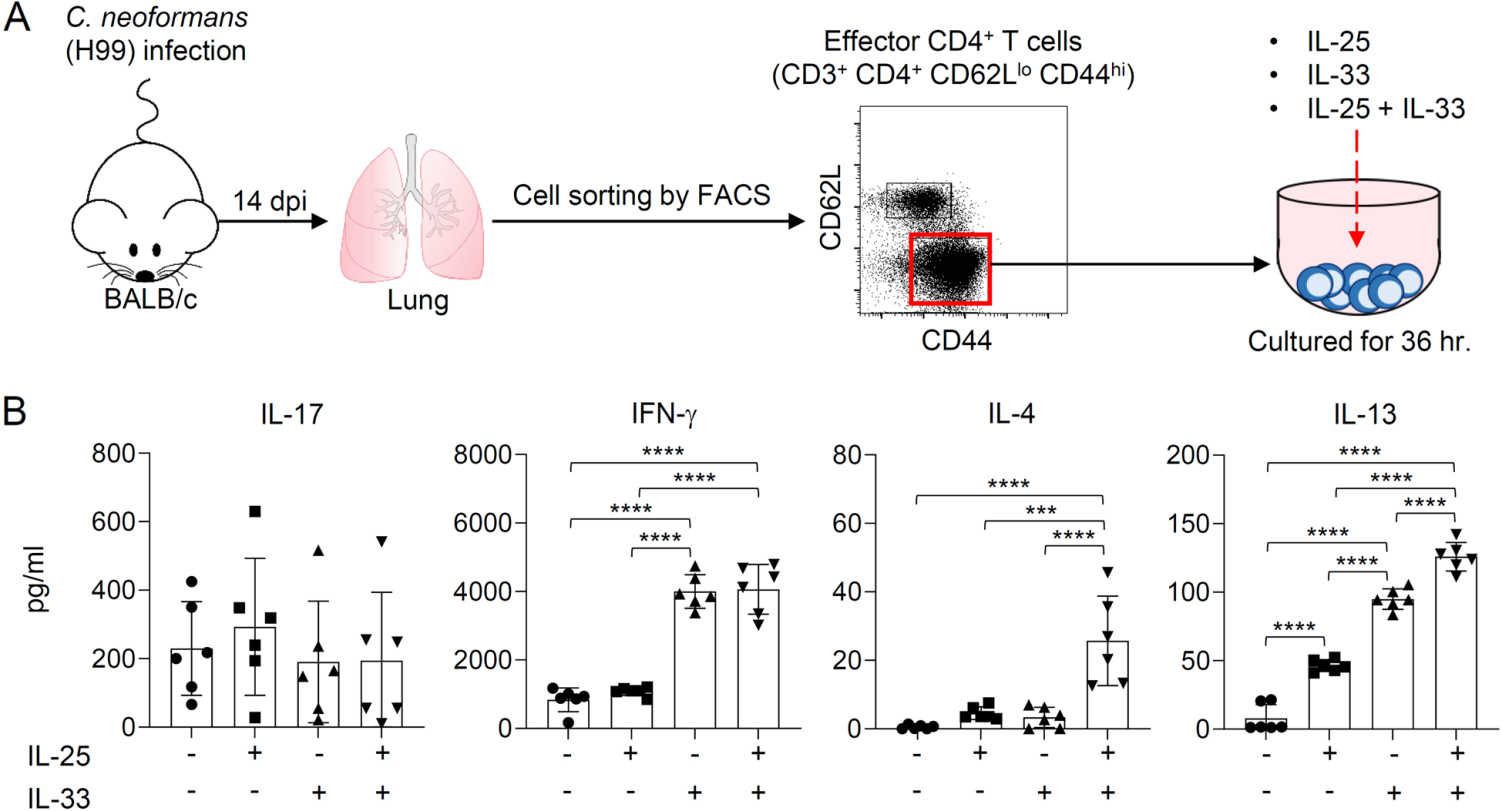
IL-25 and IL-33 preferentially induce an increase of cryptococcal-specific CD4^+^ Th2 cell responses. (**A**, **B**) BALB/c mice were treated with PBS or intranasally infected with *C. neoformans* (H99) at 5 × 10^4^ yeast cells/mouse. Pulmonary leukocytes were isolated at 14 days post-infection and used for magnetic bead purification of CD4^+^ T cells. Purified CD4^+^ T cells were stained with CD3, CD4, CD62L, and CD44, and the population of effector CD4^+^ T cells (CD62L^lo^ CD44^hi^ CD3^+^ CD4^+^) were sorted and stimulated with IL-25 and/or IL-33 at 10 ng/mL (**A**). After 36 h poststimulation, the supernatant was collected and subjected to analysis of cytokine, including IL-4, IL-13, IFN-γ, and IL-17 by ELISA (**B**). Graphs show the mean ± SD of two pooled independent experiments (n = 6 mice per group). Significance was determined using one-way ANOVA with Tukey post hoc analysis (***p < 0.001, ****p < 0.0001).

The biological effect of IL-25 and IL-33 in driving type-2 immune response was extensively demonstrated in the allergy and helminth infection model^15–18^. These cytokines function as an inducer of type-2 inflammation by promoting the production of type-2 cytokines^23^. Using our above finding, we assessed the combinatorial effect of IL-25 and IL-33 on regulating adaptive T helper cell response during the late phase of the *C. neoformans* infection. On the 14 days post-infection, the effector CD4^+^ T cells (CD3^+^CD4^+^CD44^hi^CD62L^lo^) were sorted from lungs and ex vivo stimulated with recombinant IL-25 or IL-33 or the combination of IL-25 and IL-33 (Fig. 2A). We observed no induction of IL-17 upon IL-25 or IL-33 stimulation; nevertheless, IFN-γ was enhanced when effector CD4^+^ T cells were treated with IL-33 but not IL-25 (Fig. 2B). Ex vivo stimulation of effector CD4^+^ T cells with either IL-25 or IL-33 did not enhance IL-4 production; however, it induced a significantly higher amount of IL-13 than untreated cells (Fig. 2B). Combination treatment of effector CD4^+^ T cells with IL-25 and IL-33 did not affect IL-17 and IFN-γ but synergistically enhanced the production of IL-4 and IL-13 (Fig. 2B). Our data suggest that IL-25 and IL-33, when taken together, exhibited a differential and combinatorial effect on the promotion of the production of cytokines by adaptive T helper cells during *C. neoformans* infection.

### Transcriptomic analysis revealed the effect of IL-25 and IL-33 on the biological pathways of effector CD4^+^ T cell responses during ***C. neoformans*** infection

**Figure 3:**
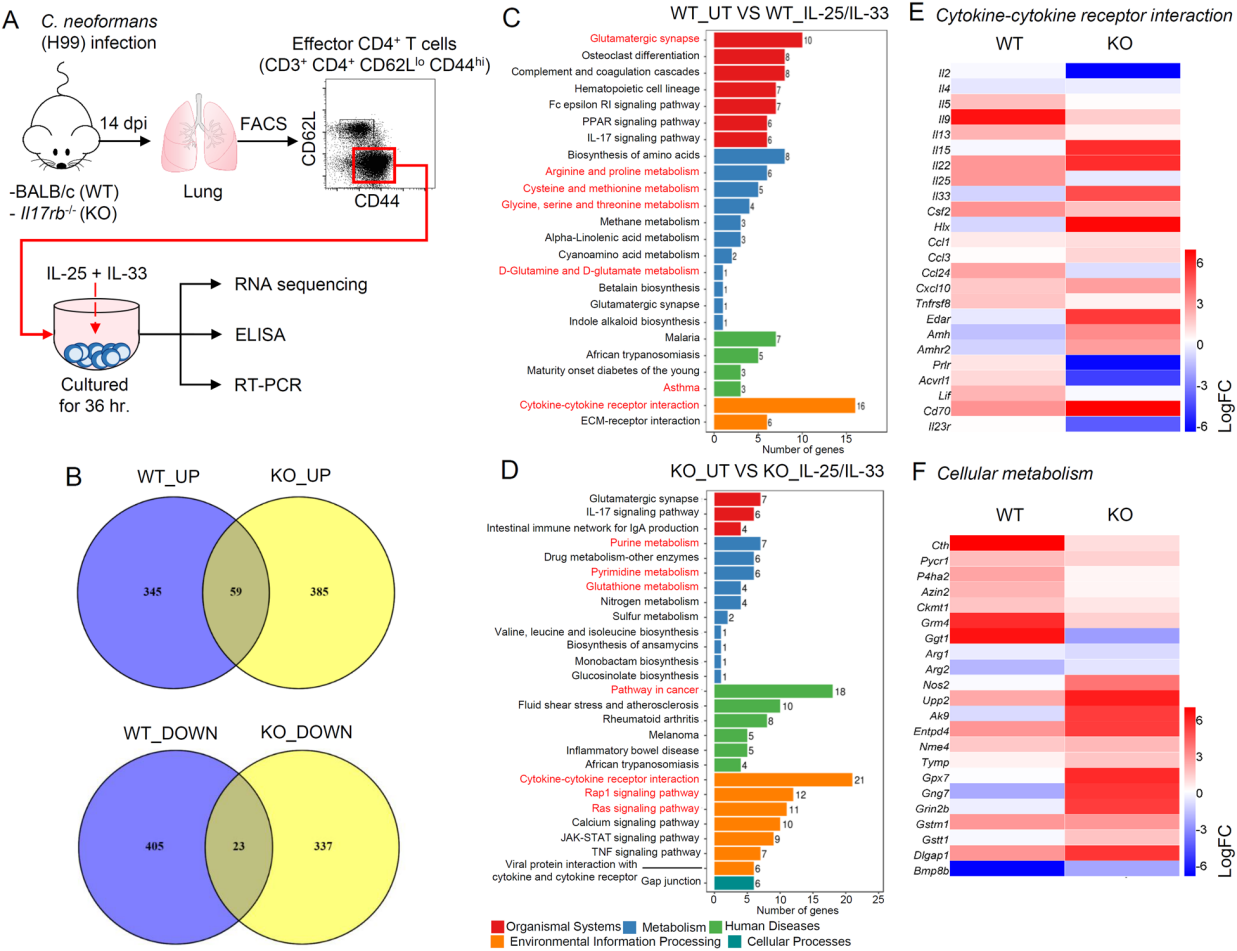
Transcriptomic analysis of cryptococcal specific-T helper cell stimulated with IL-25/IL-33. (**A**–**F**) BALB/c (WT) and *Il17rb*^−/−^ (KO) mice were treated with PBS or intranasally infected with *C. neoformans* (H99) at 5 × 10^4^ yeast cells/mouse. Lung CD4^+^ T cells were purified, and the population of effector CD4^+^ T cells (CD62L^lo^ CD44^hi^ CD3^+^ CD4^+^) was sorted and stimulated with IL-25 and IL-33 at 10 ng/mL. After 36 h poststimulation, the culture supernatant was collected for the analysis of cytokines by ELISA and stimulated cells were collected and subjected to RNA sequencing and analysis of gene expression by real-time PCR (**A**). (**B**–**F**) The transcriptome of effector CD4^+^ T cells isolated from WT and KO mice treated with IL-25/IL-33 (WT_IL-25/IL-33 and KO_IL-25/IL-33) was compared with untreated control (WT_UT and KO_UT). (**B**) Venn diagrams (plotted by BioinfoGP Venny v2.1.0) compare the upregulated and downregulated DEGs in normalized WT_IL-25/IL-33 versus KO_IL-25/IL-33 effector CD4^+^ T cells. The numbers in the overlap areas indicate the common DEGs found in the compared transcriptomes. The numbers in the non-overlap areas indicate the unique DEGs of the transcriptomes. (**C**, **D**) The bar charts of the significant unique KEGG pathways (created by clusterProfiler R package v3.8.1) observed in the normalized transcriptome of WT_IL-25/IL-33 (**C**) and KO_IL-25/IL-33 (**D**) effector CD4^+^ T cells. The y-axis represents the name of certain KEGG pathways. The x-axis refers to the number of genes enriched in the term. (**E**–**F**) Heatmap (plotted by HemI V1.0.3.7) data show the expression of genes presented Log Fold change value in the group of genes associated with cytokine-cytokine receptor interaction (**E**) and cellular metabolism (**F**). The gradient color-coded scale indicates the magnitude of changes in the gene expression levels: red indicates upregulation, and blue indicates downregulation.

To clarify the molecular mechanisms by which IL-25 and IL-33 differentially modulate adaptive T helper cell responses during *C. neoformans* infection, we sorted lung effector CD4^+^ T cells from BALB/c (wild-type; WT) and *Il17rb*^−/−^ (KO) mice after *C. neoformans* infection for 14 days. These cells were ex vivo stimulated with a combination of IL-25 and IL-33 for 36 h, and total RNA from the three independent experiments was pooled and subjected to RNA sequencing (Fig. 3A). We conducted differentially expressed gene (DEG) analysis by comparing the transcriptome data of IL-25/IL-33-treated effectors CD4^+^ T cells of WT (WT_IL-25/IL-33) and KO (KO_IL-25/IL-33) mice with the untreated cells. Data on the reading quality of four transcriptomes (Supplementary Tables S1 and S2) and the database of DEGs (Supplementary Tables S3 and S4) were provided, and the pairwise comparison between upregulated and downregulated DEGs in normalized WT_IL-25/IL-33 versus KO_IL-25/IL-33 effector CD4^+^ T cells was presented in a Venn diagram (Fig. 3B). Although a total of 59 upregulated DEGs were common to both groups, we identified 345 upregulated DEGs uniquely expressed in WT_IL-25/IL-33 and 385 upregulated DEGs in KO_IL-25/IL-33 (Fig. 3B). Indeed, a total of 23 downregulated DEGs were shared in both groups, but 405 and 337 downregulated DEGs were distinctly identified for WT_IL-25/IL-33 and KO_IL-25/IL-33, respectively (Fig. 3B).

The upregulated and downregulated DEGs were annotated to the Kyoto Encyclopedia of Genes and Genomes (KEGG) pathways to further analyze the biological pathway and the molecular network on WT and KO effector T helper cells treated with IL-25/IL-33 and the most significant pathway with false discovery rate (FDR) q-value of < 0.05 (Fig. 3C–D). Twenty-four pathways in WT_IL-25/IL-33 transcriptomes were associated with cytokine–cytokine receptor interaction, asthma, arginine metabolism, proline, cysteine, methionine, glycine, serine, threonine, glutamine, and glutamate. Conversely, 28 pathways in the KO_IL-25/IL-33 transcriptomes were associated with cytokine–cytokine receptor interaction, pathway in cancer, glutathione metabolism, purine, and pyrimidine as well as Ras/Rap1 signaling (Fig. 3C–D). These data indicated that the cytokine–cytokine receptor interaction and cellular metabolism were the major enriched pathways in response to IL-25 and IL-33 treatment of both WT and KO T helper cells.

### Heatmap and gene expression analysis revealed the combinatorial and differential effects of IL-25 and IL-33 cytokines in effector CD4^+^ T cell responses during *C. neoformans* infection

We further performed heatmap analysis to reveal each significant DEGs in the pathway of cytokine–cytokine receptor interaction and cellular metabolism of IL-25- and IL-33-treated effector CD4^+^ T helper cells purified from *C. neoformans*-infected WT and *Il17rb*^−/−^ KO mice. In response to IL-25 and IL-33 treatment, WT effector CD4^+^ T helper cells exhibited a strong induction of genes associated with type-2 immune responses (*Il5*, *Il9*, *Il13*, *Il25*, *Csf2*, and *Ccl24*), metabolism of arginine/proline (*Azin2*, *Ckmt1*, and *Pycr1*), and cysteine/glutathione (*Ggt1* and *Cth*) as well as T cell activation and survival (*Tnfrsf8*, *Prlr*, *Acvrl1*, and *Lif*) and downregulation of type-1 associated genes (*Hlx*, *Cxcl10*, and *Ccl3*) (Fig. 3E–F and Supplementary Table S5). However, the heatmap analysis of IL-25/IL-33-treated *Il17rb*^−/−^ effector CD4^+^ T helper cells showed a higher induction of *Il33* and genes associated with type-1 immune responses (*Hlx*, *Ccl1*, *Ccl3*, *Cxcl10*, and *Nos2*), T cell activation and development (*Il15*, *Il22*, and *Cd70*), metabolism of purine/pyrimidine nucleotide (*Upp2*, *Ak9*, *Entpd4*, *Nme4*, and *Tymp*), glutathione (*Gpx7* and *Gstm1*), and a lower expression of type-2 associated genes (*Il5*, *Il9*, *Il13*, *Il25*, *Csf2*, and *Ccl24*) and metabolism of arginine/proline (*Azin2* and *Ckmt1*) (Fig. 3E–F and Supplementary Table S6).

**Figure 4:**
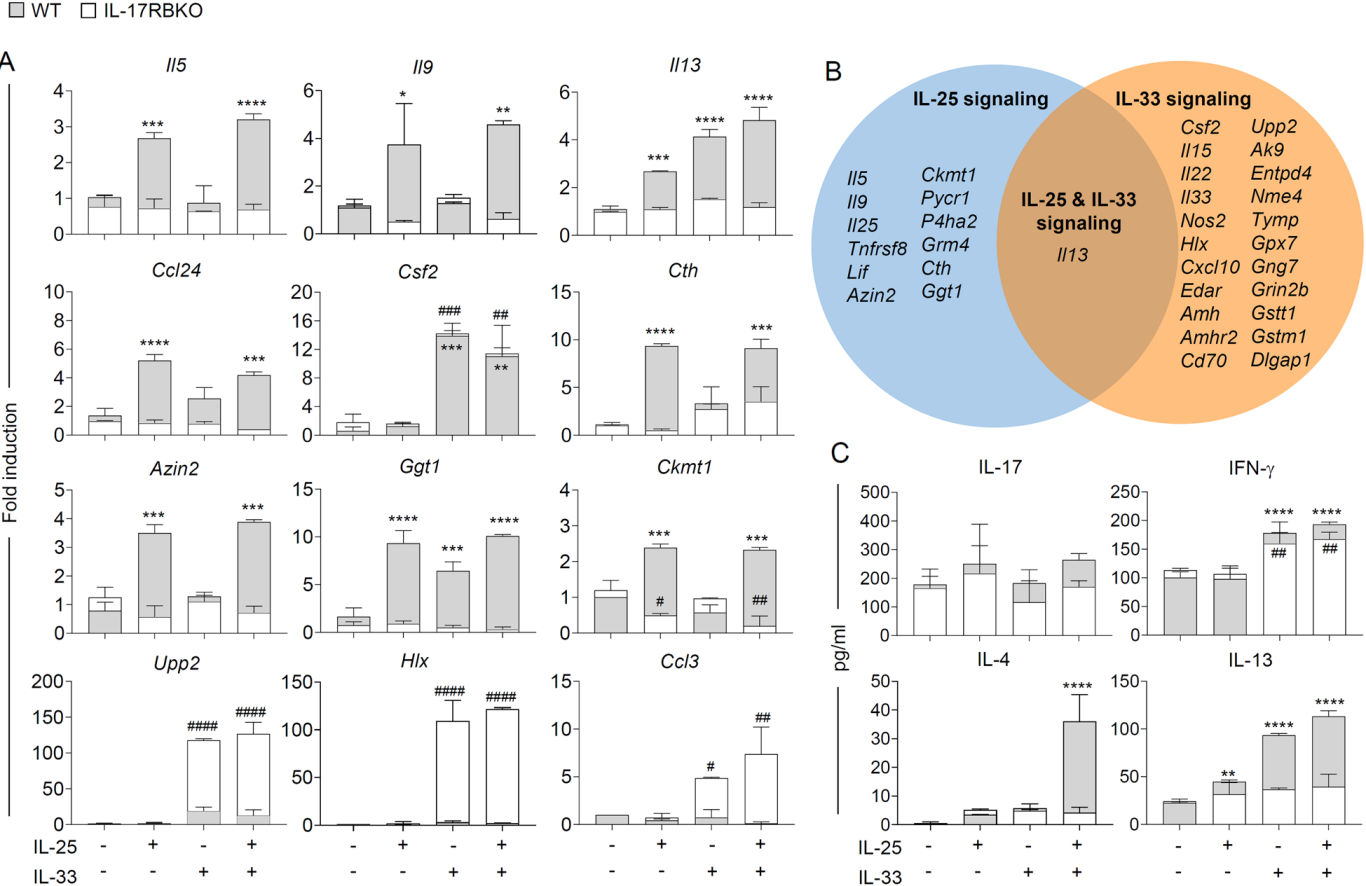
Differential and combinatorial effect of IL-25 and IL-33 in regulating pathogenic T helper cell response during *C. neoformans* infection. (**A**–**C**) BALB/c (WT) and *Il17rb*^−/−^ (KO) mice were treated with PBS or intranasally infected with *C. neoformans* (H99) at 5 × 10^4^ yeast cells/mouse. Lung CD4^+^ T cells were purified, and the population of effector CD4^+^ T cells (CD62L^lo^ CD44^hi^ CD3^+^ CD4^+^) was sorted and stimulated with IL-25 and/or IL-33 at 10 ng/mL. After 36 h poststimulation, the stimulated cells were collected and subjected to analysis of gene expression by real-time PCR (**A**–**B**) (**A**) Verification analysis of genes significantly detected in the normalized transcriptome of WT_IL-25/IL-33 and KO_IL-25/IL-33 effector CD4^+^ T cells, including *Il5, Il9, Il13, Ccl24, Csf2, Cth, Ggt1, Ckmt1*, *Azin2, Hlx, Ccl3,* and *Upp2* by real-time PCR. Expression levels of the target genes were normalized to endogenous actin (*Actb*) transcript levels. The sample’s relative quantification (fold induction) was calculated using the untreated control as a baseline. (**B**) Venn diagrams indicate a summary of genes within cryptococcal-specific T helper cells regulated by IL-25, IL-33, and IL-25/IL-33 signaling. (**C**) The culture supernatant was collected for the analysis of cytokines including IL-17, IFN-γ, IL-4, and IL-13 by ELISA. Graphs show the mean ± SD of two pooled independent experiments (n = 6 mice per group). Significance was determined using two-way ANOVA with Tukey’s multiple comparison test (WT *p < 0.05, **p < 0.01, ***p < 0.001, ****p < 0.0001; KO #*p* < 0.05, ##*p* < 0.01, ###*p* < 0.001).

Based on the transcriptomic results, we further verified the effect of IL-25 and IL-33 by treating effector CD4^+^ T cells isolated from day 14–infected WT and *Il17rb*^−/−^ mice with recombinant IL-25 or IL-33 or the combination of IL-25 and IL-33. After 36 h, the stimulated cells were collected for gene expression analysis. The most significantly upregulated and downregulated DEGs were selected as representative genes of each pathway, including type-2 immune responses (*Il5*, *Il9*, *Il13*, *Ccl24*, and *Csf2*), metabolism of cysteine/glutathione (*Cth* and *Ggt1*), arginine/proline (*Azin2 and Ckmt1*), and purine/pyrimidine nucleotide (*Upp2*), as well as type-1 immune response (*Hlx* and *Ccl3*). We observed the significant upregulation of *Il5*, *Il9*, and *Ccl24* in WT effector CD4^+^ T cells treated with IL-25 but not IL-33 (Fig. 4A). Interestingly, WT and *Il17rb*^−/−^ effector CD4^+^ T cells in response to IL-33, but not IL-25, enhanced the expression of *Csf2* genes that encodes granulocyte–macrophage colony-stimulating factor. By comparing gene expression analysis between WT and *Il17rb*^−/−^ effector CD4^+^ T cells, the synergistic effect of IL-25 and IL-33 was only detected in enhancing IL-13 expression (Fig. 4A,B). The expression levels of *Cth*, *Ckmt1*, and *Azin2* genes, involved in the amino acid metabolism, were significantly enhanced upon IL-25 but not IL-33 stimulation in WT effector CD4^+^ T cells (Fig. 4A). Treatment with either IL-25 or IL-33 enhanced the expression of *Ggt1*, genes involved in the metabolism of glutathione, indicating the function of both cytokines in regulating T cell response to oxidative stress during *C. neoformans* infection. Furthermore, the significant upregulation of genes associated with Th1-type immune responses (*Hlx* and *Ccl3*) and purine/pyrimidine nucleotide metabolism (*Upp2*) were observed only in *Il17rb*^−/−^ effector CD4^+^ T cells stimulated with IL-33 but not for IL-25 (Fig. 4A,B). Consistent with the transcriptome data, *Il17rb*^−/−^ effector CD4^+^ T cells stimulated with IL-25 or IL-33 exhibited reduced IL-4 and IL-13 secretion when compared to WT CD4^+^ T cells (Fig. 4C). The level of IFN-γ was enhanced in *Il17rb*^−/−^ effector CD4 + T cells stimulated with IL-33 but not IL-25 (Fig. 4C). When taken together, IL-25 and IL-33 had a differential and combinatorial effect on modulating effector T helper cells during cryptococcal disease progression. In pulmonary cryptococcosis, signaling of IL-25 is required for amplifying effector Th2-type immune responses and downregulating Th1-type immune responses. IL-33 not only acts synergistically to augment type-2 immune responses but also has a unique role in activating the metabolism of purine/pyrimidine nucleotide to promote T cell proliferation during cryptococcal disease progression.

### The pulmonary IL-17RB^+^ST2^+^ effector CD4^+^ T cells were associated with cryptococcal virulence and cryptococcal infection in the brain

**Figure 5:**
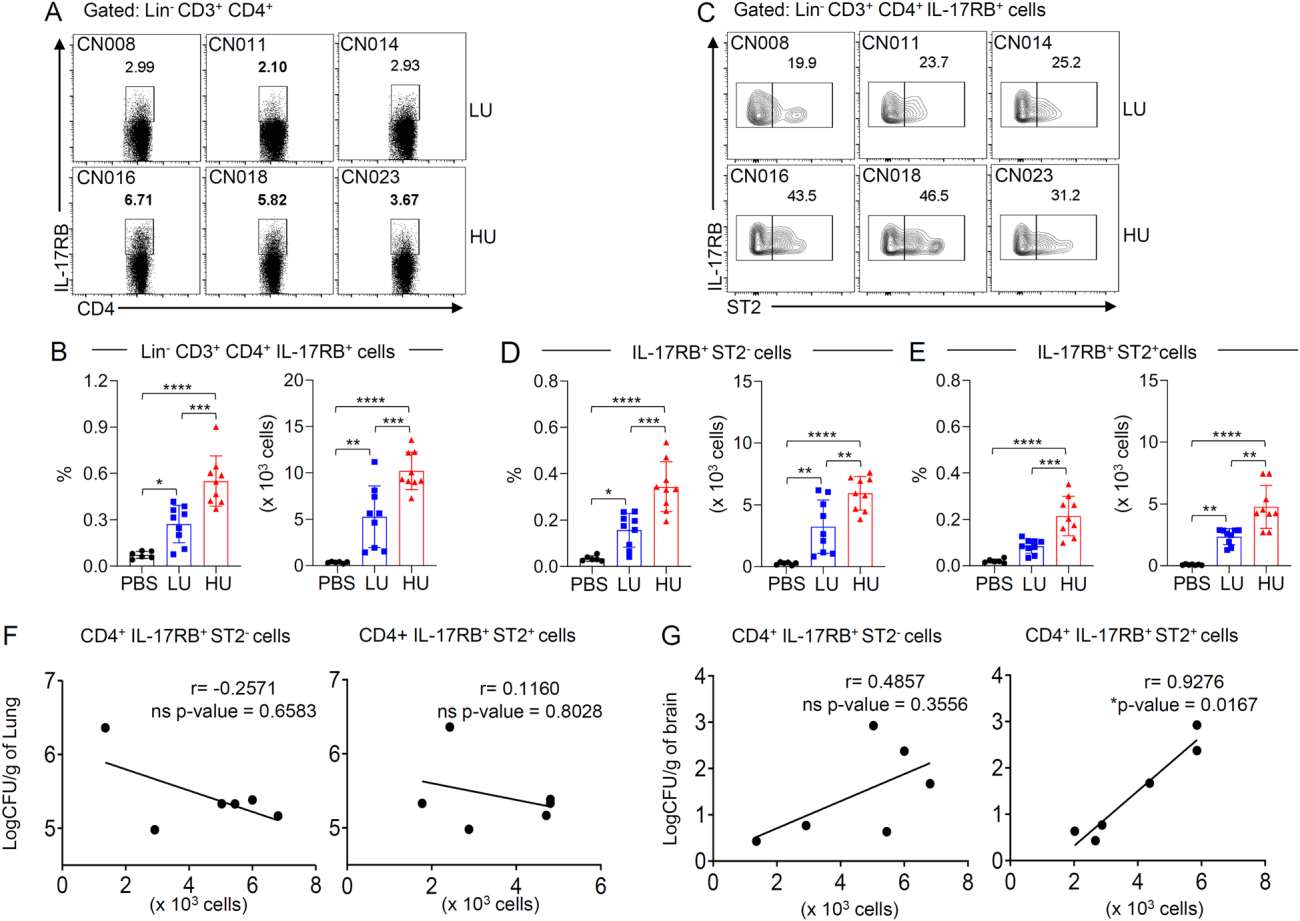
High-virulence strain of *C. neoformans* potentiates the induction of lung IL-17RB/ST2 co-expressing T cells population associated with cryptococcal brain dissemination. (**A**–**G**) BALB/c mice were treated with PBS or intranasally infected with *C. neoformans* clinical isolates with HU and LU (three isolates per group) at 5 × 10^4^ yeast cells/mouse. (**A**–**E**) Pulmonary leukocytes were isolated 14 days post-infection and stained with fluorescence-conjugated Ab to lineage markers (Lin; CD11b, CD11c, B220, CD19, DX5, Gr.1, and CD8), CD3, CD4, IL-17RB, and ST2. The population of T helper cells within the lung was analyzed for the expression of IL-17RB and ST2 by flow cytometry. (**A**, **B**) Representative plots (**A**) and frequencies depicted as the percentages and absolute count (**B**) of the total IL-17RB-expressing T helper cell population (Lin^-^ CD3^+^ CD4^+^ IL-17RB^+^). (**C**–**E**) Phenotypic analysis of IL-17RB-expressing T helper cell for the expression of ST2. The gated (**C**) and frequencies of the IL-17RB^+^ ST2^-^ (**D**) and IL-17RB^+^ ST2^+^ (**E**) T helper cells are depicted as the percentages and absolute count. Graphs show individual mice and mean ± SD and represent two pooled independent experiments (n = 6 mice per group). Significance was determined using one-way ANOVA with Tukey post hoc analysis (*p < 0.05, **p < 0.01, ***p < 0.001, ****p < 0.0001). (**F**–**G**) The analysis of the correlation of lung (**F**) and brain (**G**) CFU with the absolute number of IL-17RB^+^ ST2^-^ and IL-17RB^+^ ST2^+^ T cells within the lung of infected mice. Correlation coefficients (*r*) and *p* values are given for each correlation. (*p < 0.05. ns, not significant).

Related studies indicated that *C. neoformans* clinical isolates that were more easily phagocytosed by macrophages (termed high-uptake *C. neoformans*) were associated with higher fungal burden in the brain^24,25^. Our previous study also suggested that *C. neoformans* with a high-uptake rate (HU) exhibited an increasing ability to disseminate to the brain than those with a low-uptake rate (LU), and this ability was associated with elevated type-2 cytokine production^26^. We thus further characterized the population of IL-17RB^+^ST2^+^ effector CD4^+^ T cells in the lungs of mice infected with *C. neoformans* strains with different virulence (LU, isolates showed a lower phagocytosis rate: CN008, CN011, and CN014; and HU, isolates showed a higher phagocytosis rate: CN016, CN018, and CN023) for 14 days. In comparison to the PBS-treated mice, we found a higher number of IL-17RB^+^ CD4^+^ T cells in the lungs of mice that were infected with HU than in those infected with LU (Fig. 5A,B). Further characterization of ST2 expression in these cells showed higher frequencies of both IL-17RB^+^ ST2^-^ and IL-17RB^+^ ST2^+^ effector CD4^+^ cells in the lungs of mice infected with HU than in those infected with LU strains (Fig. 5C–E). The number of these cells was then associated with the fungal burdens in the lungs and brain. Indeed, there was no association between the frequency of IL-17RB^+^ ST2^−^ or IL-17RB^+^ ST2^+^ effector CD4^+^ cell and lung fungal burdens (Fig. 5F and Supplementary Table S8). Interestingly, the frequencies of IL-17RB^+^ ST2^+^ but not IL-17RB^+^ ST2^−^ effector CD4^+^ T cells were strongly correlated with the brain fungal burden (r = 0.9429, *p = *0.0167) (Fig. 5G and Supplementary Table S8). Our data thus indicate that high cryptococcal virulence promoted the expression of IL-17RB and ST2 by effector CD4^+^ cells. During later stages of infection, these cells respond to IL-25 and IL-33 to promote the activation of pathogenic T helper cells that favor fungal survival and support cryptococcal dissemination to the brain.

## Discussion

Previous studies indicated that the epithelial cell-derived cytokines IL-33 and IL-25 promote a pathogenic type-2 environment during *C. neoformans* infection^19–22^. Nevertheless, how these cytokines cooperate in regulating adaptive T helper cell response during *C. neoformans* infection has not yet been clarified. In this study, we characterized the expression patterns of IL-17RB and ST2 on T helper cells. We investigated their cooperative functions by analyzing transcriptome patterns of IL-25- and IL-33-treated effector CD4^+^ T cells of WT and *Il17rb*^−/−^ mice after being infected with *C. neoformans*. The IL-17RB-expressing T helper cells were induced and largely co-expressed with ST2 in pulmonary *C. neoformans* infection at the late infection phase. The comparative transcriptomic analysis revealed a cooperative effect of IL-25 and IL-33 on enhancing IL-13 production. IL-25 signaling had a distinct effect in upregulating the expression of type-2 cytokines and chemokines. Conversely, IL-33 was uniquely involved with T cell survival and inflammatory responses of effector T helper cells during cryptococcal disease progression. Indeed, the frequencies of IL-17RB and ST2-co-expressing effector T helper cells were associated with *C. neoformans* infection with higher virulence and strongly correlated with cryptococcal brain dissemination.

The epithelial cell-derived cytokines IL-25 and IL-33 can potentiate type-2 immune responses by activating both ILC2 and Th2 cells in allergy and helminth infection^15–18^. Previous studies indicated that pulmonary IL-25 was upregulated in response to *C. neoformans* infection later than IL-33^20^, indicating that these cytokines may preferentially function in promoting cryptococcal disease pathogenesis at different infection stages. For the pulmonary *C. neoformans* infection, adaptive T helper cells were likely to be the main target of these cytokines in mediating the pathogenesis of cryptococcal diseases^19,20^. In this study, we further analyzed the expression of the cognate receptor for IL-25 and IL-33 on pulmonary CD4^+^ T cells during pulmonary *C. neoformans* infection. We found that most IL-17RB^+^ CD4^+^ T cells were co-expressed with ST2, particularly at the late phase of infection. IL-25 and IL-33 may likely have a differential and cooperative effect on T helper cells in promoting pathogenic T helper cell response. Recent studies described a specific subpopulation of Th2 cells, called pathogenic T helper 2 cells (Tpath2 cells), which are capable of causing allergic asthma and chronic allergic dermatitis by producing excessive amounts of type-2 cytokines, particularly IL-5 and IL-13^27–29^. Indeed, co-treatment of effector CD4^+^ T cells with IL-25 and IL-33 greatly enhanced the secretion of IL-13. Unlike IL-25, IL-33 potentiated the production of Th1 cytokine, IFN-γ, indicating the differential effect of IL-33 in promoting both Th1 and Th2 immune response during *C. neoformans* infection. The comparative transcriptomic analysis of lung effector T helper cells of *C. neoformans*-infected mice revealed a strong induction of type-2 cytokine and chemokine genes, including *Il5*, *Il9*, *Il13*, *Csf2*, and *Ccl24* after IL-25 and IL-33 co-treatment, highlighting the importance of these cytokines in amplifying type-2 inflammatory response. Without IL-25 receptor signaling, IL-25 and IL-33-treated effector T helper enhanced the production of IFN-γ along with the expression of IFN-γ-associated transcription factor *Hlx* and type-1 chemokines, including *Ccl1*, *Ccl3*, and *Cxcl10*. Unlike IL-25, IL-33 was known to have pleiotropic effects on CD4^+^ T cell differentiation and effector function by promoting Th1 and Th2 immune responses depending on the cytokine environment in damaged tissue^30^. The expression of ST2 was required for Th1 cell expansion and function during LCMV infection^31,32^. It is also likely that deficiency of IL-17RB may alter effector T helper cell phenotypes toward inflammatory Th1 cells; thus, IL-33 had a distinct effect on potentiating type-1 associated genes. Further investigation using single-cell RNA sequencing may reveal the cellular diversity and dynamic inflammatory processes of effector T helper cells during C. *neoformans* infection. IL-25 and IL-33, when taken together, had differential and combinatorial effects on functional cytokine and chemokine response of adaptive T helper cells during *C. neoformans* infection.

KEGG pathway enrichment analysis of differential genes revealed that IL-25 signaling preferentially upregulated genes in the pathways associated with L-arginine metabolisms such as *Azin2* (antizyme inhibitor 2)*, Ckmt1* (creatine kinase mitochondrial 1), *Pycr1* (pyrroline-5-carboxylate reductase 1), and anti-oxidant pathway (*Cth* [cystathionine gamma-lyase] and *Ggt1* [gamma-glutamyltransferase 1]). *Azin2*, *Ckmt1*, and *Pycr1* are involved with the conversion of arginine to polyamine and proline that promotes T cell proliferation and survival^33–40^. *Cth* and *Ggt1* are genes encoding the enzymes in cysteine metabolism, a precursor for the anti-oxidative substance glutathione^41,42^. IL-33 upregulated genes in the pathways of nucleotide metabolism, including *Upp2* (uridine phosphorylase 2), *Ak9* (adenylate kinase 9), *Entpd4* (ectonucleoside triphosphate diphosphohydrolase 4), *Nme4* (NME/NM23 nucleoside diphosphate kinase 4), and *Tymp* (thymidine phosphorylase). These enzymes were associated with the pathway that utilized purine and pyrimidine nucleotide as an energy source for the survival of the cell^43–51^. Hence, signaling of IL-25 and IL-33 on CD4^+^ T helper cells not only affects different cytokine and chemokine profiles but also influences different pathways of cell activation, survival, and homeostasis. The importance of IL-25- and IL-33-responsive T helper cells was emphasized by finding higher frequencies of IL-17RB^+^ST2^+^CD4^+^ T helper cells in the lung of mice infected with high-virulence *C. neoformans* than those infected with the low-virulence and a positive correlation between these population and brain fungal burden. Although IL-25 and IL-33 have a collaborative and differential effect on effector T helper cells, infection with high-virulence *C. neoformans* may alter CD4^+^ T helper to express both IL-17RB and ST2 for the cooperative effect of IL-25 and IL-33 in mediating amplification of pathogenic Th cell response to support cryptococcal brain dissemination.

In summary, we presented a population of IL-17RB^+^ST2^+^ pathogenic Th cells that associates with cryptococcal virulence and disease progression. During *C. neoformans* infection, pulmonary IL-25 and IL-33 regulate effector T helper cells by having a combinatorial effect on IL-13 production and a differential effect on cytokine, chemokine, and pathways of cell survival and metabolism. Furthermore, it will be interesting to not only utilize the adjunctive therapy with IFN-γ but also inhibit Th2-type cytokine initiator IL-25 and IL-33 to promote protective immunity during *C. neoformans* infection.

## Materials and methods

### Mice

BALB/c female mice, aged 6–8 weeks, were purchased from Nomura Siam International Co., Ltd, Thailand. *Il17rb*^−/−^ mice were bred and housed under specific pathogen-free conditions in the animal facility of Thammasat University. Mice with matching age and sex were used in all experiments. Mice were euthanized at the indicated time using controlled gradual displacement of CO_2_ in accordance with the Institutional Animal Care and Use Committee and the American Veterinary Medicine Association guidelines. The Thammasat University Animal Care and Use Committee reviewed and approved all experimental animal protocols (001/2021). The study was conducted based on the ARRIVE guidelines.

### Culture of *Cryptococcus*

Both references (H99) and all clinical isolates of *C. neoformans* LU (isolates showed a lower phagocytosis rate: CN008, CN011, and CN014) and HU (isolates showed a higher phagocytosis rate: CN016, CN018, and CN023)^26^ (Supplementary Table S7) were obtained from the Department of Microbiology, Faculty of Medicine, Siriraj Hospital, Mahidol University and maintained at the Department of Medical Technology, Faculty of Allied Health Sciences, Thammasat University. Before the experiments, these fungi were retrieved from the culture stock and cultured on Sabouraud dextrose agar (SDA) (HiMedia) at room temperature (25–29 °C) for 48 h. Then, a single colony of yeast cells was cultured in Sabouraud dextrose broth (BD Difco™) at 37 °C with shaking at 200 rpm for 24 h before infection. Thammasat Institutional Review Board ethically approved this study under certificate number 023/2564 (Exempt).

### Intranasal inhalation of *Cryptococcus*

The murine pulmonary *C. neoformans* infection model was performed as previously described^20^. Briefly, BALB/c or *Il17rb*^−/−^ mice were anesthetized and intranasally treated with either PBS or *C. neoformans* suspension at 5 × 10^4^ cells/mouse. At the studied time, mice were euthanized by inhalation of CO_2_. To analyze fungal organ burden, the lungs and brains of mice were aseptically collected, weighed, and homogenized in sterile PBS with 1% penicillin and streptomycin, followed by plating on SDA at various dilutions. The organ CFU was counted and calculated after incubation at room temperature for 48 h.

### Lung leukocyte isolation and flow cytometric analysis

The isolation of leukocytes within the lung was performed as previously described^20,52,53^. Briefly, lungs were minced using sterile 100-mm nylon mesh in the complete medium of RPMI (RPMI 1640 containing 10% fetal bovine serum, 1% L-glutamine, and 1% penicillin and streptomycin). After removing contaminated red blood cells, fungus, and debris, a hemocytometer counted the single-cell suspension in trypan blue. Then, it was stained with fluorochrome-conjugated antibodies, including PerCP-Cy5.5–conjugated-anti-lineage (Lin) markers CD11b (M1/70), CD11c (HL3), Gr.1 (RB6-8C5), CD49b (DX5), CD8 (53–6.7), B220 (RA3-6B2), CD335 (NKP46, 29A.4), allophycocyanin (APC)-conjugated-anti-IL-17RB (9B10), eFluor^®^506-conjugated anti-CD4 (RM4-5), PE-Cy7–conjugated anti-CD3e (145-2C11), biotinylated anti-T1/ST2 (RMST2-33), and Brilliant Violet 421–labeled streptavidin from BioLegend. In some experiments, phycoerythrin (PE)-conjugated anti-CD62L (MEL-14), Pacific Blue–conjugated anti-CD44 (IM7), and PerCP-Cy5.5–conjugated anti-T1/ST2 (RMST2-33) were used. The addition of the anti-CD16/32 antibody was used to block Fc receptors. The debris and dead cells were removed by the gating of forward versus side scatter gating and 7-AAD staining. The population of T helper cells (Lin^-^ CD3^+^ CD4^+^) and naïve (CD62L^hi^ CD44^lo^ CD3^+^ CD4^+^) or effector CD4^+^ T cell (CD62L^lo^ CD44^hi^ CD3^+^ CD4^+^) were further characterized for the expression of the receptor for IL-25 and IL-33 (IL-17RB and ST2, respectively) using a BD FACSLyric cytometer (BD Biosciences, San Jose, CA), and the data were analyzed by using the FlowJo Software (Treestar).

For sorting of effector CD4^+^ T cells, pulmonary leukocytes isolated from the lung of *C. neoformans*-infected WT or *Il17rb*^−/−^ mice at 14 days post-infection were further incubated with rat-anti-mouse CD4 microbeads (L3T4) to purify for CD4^+^ T cells by positive selection using a magnetic purification system (Miltenyi Biotec). The number of CD4^+^ T cells was determined by using a trypan blue exclusion assay followed by blocking with anti-CD16/CD32 and staining with the following fluorochrome-conjugated antibodies: Alexa Fluor^®^ 488 conjugated anti-CD3e (145-2C11), PE-conjugated anti-CD62L (MEL-14), PerCP-Cy5.5-conjugated anti-CD4 (RM4-5), and APC-conjugated anti-CD44 (IM7) from BioLegend. The debris and dead cells were removed by gating forward versus side scatter gating and 7-AAD staining. The population of effector CD4^+^ T cells (CD62L^lo^ CD44^+^ CD3^+^ CD4^+^) was sorted using a FACSAria III (BD Biosciences, San Jose, CA).

### Ex vivo stimulation of effector CD4^+^ T cells

Purified effector CD4^+^ T cells isolated from the lung of day 14–infected WT or *Il17rb*^−/−^ mice at 2 × 10^5^ cells were stimulated with recombinant IL-25 and/or IL-33 (R&D Systems) at 10 ng/mL in 96-well culture plate at 37 °C, 5% CO_2_^54–56^. After 36 h, the culture supernatant and stimulated cells were collected to analyze cytokine production via enzyme-linked immunosorbent assay (ELISA) and gene expression via RNA sequencing or real-time polymerase chain reaction (real-time PCR), respectively.

### Transcriptomic analysis of effector CD4^+^ T cells

#### mRNA library constructing and sequencing

The effector CD4^+^ T cells isolated from the lung of *C. neoformans* H99-infected WT or *Il17rb*^−/−^ mice (KO) (three mice per group) with untreated (WT_UT and KO_UT) or treated with IL-25 and IL-33 (WT_IL-25/IL-33 and KO_IL-25/IL-33) were pooled from three independent experiments and total RNA was extracted by using RNeasy^®^ Plus Mini kit (Qiagen Ltd.) according to the manufacturer’s instructions. Then, the preparation and sequencing of the cDNA library were performed by Azenta Life Sciences (www.azenta.com). Briefly, the total RNA at 1 µg was used to generate the cDNA library. The poly(A) mRNA was isolated and fragmented using Oligo(dT) beads and divalent cations. The random primers were used to generate double-stranded cDNA. The purified double-stranded cDNA was then modified to add adaptors at both ends by adding dA-Tailing and T-A ligation. The DNA Clean Beads were used to selection of adaptor-ligated DNA based on the size, which was then amplified by PCR using primers P5 and P7. After validation of the PCR products, the libraries with different indexes were multiplexed and loaded on an Illumina HiSeq instrument for sequencing according to the manufacturer’s instructions. The quality of raw sequencing data was evaluated by using QPhred (Supplementary Table S1), and the sequencing data with high-quality or clean reads were filtered by Cutadapt (version 1.9.1) and were used for further bio-informatic analysis (Supplementary Table S2). The RNA sequencing data are deposited in the database of NCBI Gene Expression Omnibus (GEO) (https://www.ncbi.nlm.nih.gov/geo/query/acc.cgi?acc=GSE229703) under accession number GSE229703.

#### Gene mapping and expression

The *Mus musculus* reference genome GRCm39.104 was utilized as a reference genome to align with the high-quality paired-end reads using Hisat2 (v2.0.1). To annotate the reads based, NCBI BLAST v2.2.28 was used with the expected value of 1.0 × 10^−10^. The gene abundance in each transcript was counted and then normalized by fragment per kilobases per million mapped reads using HTSeq v0.6.1.

#### Analysis of DEGs and clustering

Bioconductor EdgeR software (V3.4.6) was used to evaluate the read counts from each sequenced library which was adjusted by using one scaling normalized factor. The analysis of DEGs was performed using the Bioconductor package DESeq2 (V1.6.3), a model based on the negative binomial distribution. The assessment of dispersion and logarithmic fold changes incorporate data-driven prior distributions, Padj of genes was set to < 0.05 to detect DEGs. DEGs with upregulation and downregulation criteria were determined at > 2 log_2_(fold change) and <  − 2 log_2_(fold change), respectively. *P* < 0.05 was the threshold for indicating significant differential expression. The pairwise comparison of two sets of DEGs was performed, including (1) effector CD4^+^ T cell of *C. neoformans* H99-infected wild-type mice of the untreated compared to those treated with IL-25 and IL-33 (WT_UT VS WT_IL-25/IL-33) and (2) effector CD4^+^ T cell of *C. neoformans* H99-infected *Il17rb*^−/−^ mice of the untreated in comparison to those treated with IL-25 and IL-33 (KO_UT VS KO_IL-25/IL-33) using likelihood ratio testing (glmLRT); DEGs from edgeR analysis were deemed significant with an FDR of ≤ 0.05. The clustering diagram of DEGs and log_2_FC was generated by Heatmap Illustrator (HemI V1.0.3.7), and the Venn diagram was plotted by BioinfoGP Venny v2.1.0.

#### GO and KEGG signaling pathways analysis

The uniquely expressed DEGs observed in the transcriptome of effector CD4^+^ T cell were categorized into molecular function, cellular components, and biological process annotated by Gene Ontology (GO). GOSeq (v1.34.1) was used to identify GO terms that annotate a list of enriched genes. The KEGG database^57–59^ was used to identify the biological pathways of the unique DEGs. The FDR-corrected *p*-value of < 0.05 indicates the significance of GO term enrichment and KEGG pathway correlation.

### Real-time RT-PCR

Total RNA of the effector CD4^+^ T cells sorted from the lung of *C. neoformans* H99-infected WT or *Il17rb*^−/−^ mice (KO) (three mice/group) untreated or treated with recombinant IL-25 and/or IL-33 was extracted using TRIzol reagent according to the manufacturer’s instructions. cDNA was synthesized from the RNA template using the reaction containing oligo-dT and RevertAid Reverse Transcriptase (Thermo Scientific™). To verify the effector CD4^+^ T cell transcriptomic result, cDNA samples were amplified using iTaq Universal SYBR Green Supermix (Bio-Rad Laboratories). Expression levels of target genes were normalized to endogenous actin (*Actb*) transcript levels, and the relative quantification of samples was compared with untreated control serving as the baseline^33^. The sequences of the primers used were as follows: *Il5* forward: 5′-CGCTCACCGGCTCTGTTG- 3′ and reverse: 5′-CCAATGCATAGCTGGTGATTTTT-3′; *Il9* forward: 5′-CATCAGTGTCTCTCCGTCCCAACTGATG- 3′ and reverse: 5′-GATTTCTGTGTGGCATTGGTCAG-3′; *Il13* forward: 5′-GCTTATTGAGGAGCTGAGCAACA-3′ and reverse: 5′-GCTTATTGAGGAGCTGAGCAACA-3′; *Ifng* forward: 5′- GATGCATTCATGAGTATTGCCAAGT-3′ and reverse: 5′-GTGGACCACTCGGATGAGCT-3′; *Csf2* forward: 5′-TCACGTTGAATGAAGAGGTAGAAA-3′ and reverse: 5′-CGTAGACCCTGCTCGAATATC-3′; *Ccl24* forward: 5′-CTCCAGAAGGCCCTCAGACTAC-3′ and reverse: 5′-GGGTCTTCATTGCGGTGG-3′; *Cth* forward: 5′-TTTATCCTGGGCTACCCTCT-3′ and reverse: 5′-CAGAGCACCCTTGATGTAGAAA-3′; *Ckmt1* forward: 5′-GAGGGATCTGGCACAACAAT-3′ and reverse: 5′-TCATGTTGCCGCCTTTCT-3′; *Ggt1* forward: 5′- AACAGAAGGCACTGACGTATC-3′ and reverse: 5′-CCTGAGACACATCGACAAACT-3′; *Azin2* forward: 5′-GTTTCAGATGGGCGAGGAG-3′ and reverse: 5′-TACTGAGGCCATCTCTTCAAATC-3′; *Hlx* forward: 5′- AGTTAAGGAAGGCAACACTCTG-3′ and reverse: 5′- GAAGAACTGTCCCGCTGAAG-3′; *Ccl3* forward: 5′-AAGGTCTCCACCACTGCCCTTG-3′ and reverse: 5′-CTCAGGCATTCAGTTCCAGGTC-3′; *Upp2* forward: 5′-GCTTGACAAAGAACTGGCTAATG-3′ and reverse: 5′-GCGACCTTGGCCTTCATAA-3; *Actb* forward: 5′-GACGGCCAGGTCATCACTATTG-3′ and reverse: 5′-AGGAAGGCTGGAAAAGAGCC-3′.

### Statistical analysis

All the experiments in this study were performed two to three times. The unpaired t-test (two-tailed) and one-way and two-way analysis of variance (ANOVA) with Turkey’s post hoc analysis were used to analyze the statistical data presented as mean values ± SD analyzed using GraphPad Prism 9 software. Statistical significance is considered based on *p* < 0.05.

## Supplementary Information

Supplementary Tables.

Supplementary Table 3.

Supplementary Table 4.

## Data Availability

All RNA-seq data have been deposited in NCBI’s Gene Expression Omnibus (GEO) (https://www.ncbi.nlm.nih.gov/geo/query/acc.cgi?acc=GSE229703) under accession number GSE229703.

## Abbreviations

WT: Wild type
UT: Untreated
IL-17RB: Interleukin-17 receptor B
ST2: Suppression of Tumorigenicity 2
Th cells: T helper cells
DEGs: Differentially expressed genes
GO: Gene ontology
KEGG: Kyoto encyclopedia of gene and genome
