# Supplementary material for: Differential and cooperative effects of IL-25 and IL-33 on T helper cells contribute to cryptococcal virulence and brain infection
Source: Sci Rep. 2023 Jun 19;13:9895. doi: 10.1038/s41598-023-37158-1 (PMC10279717; doi:10.1038/s41598-023-37158-1)
Supplement: Supplementary file 1 — Supplementary Tables. [file 41598_2023_37158_MOESM1_ESM.pdf]

Differential and cooperative effects of IL-25 and IL-33 on T helper cells contribute to cryptococcal virulence and brain infection

Adithap Hansakon[1, 2], Siranart Jeerawattanawart[1, 3], Pornpimon Angkasekwinai[1, 4*]

[1]Department of Medical Technology, Faculty of Allied Health Sciences, Thammasat University, Pathum Thani 12120 Thailand.

[2]Chulabhorn International College of Medicine, Thammasat University, Pathum Thani 12120 Thailand.

[3]Graduate Program in Biomedical Science, Faculty of Allied Health Sciences, Thammasat University, Pathum Thani 12120 Thailand

[4]Research Unit in Molecular Pathogenesis and Immunology of Infectious Diseases, Thammasat University, Pathum Thani 12120, Thailand

Email addresses

Adithap Hansakon: Hans-Adi@tu.ac.th, adithap_tumna@hotmail.com

Siranart Jeerawattanawart: siranart@hotmail.com

Pornpimon Angkasekwinai: upornpim@tu.ac.th, p.akswn@gmail.com

*** Correspondence:** Pornpimon Angkasekwinai, upornpim@tu.ac.th, p.akswn@gmail.com

Running title:

The effect of IL-25 and IL-33 combination on T helper cell response to *C. neoformans* infection

Supplementary Table S1. The summary of raw data statistics of stimulated effector CD4$^+$ T cell isolated from lung of day 14 *C. neoformans* H99-infected wild-type and *Il17rb*$^{-/-}$ mice.

Sample name*	length	Raw reads	Raw bases	Q20 (%)	Q30 (%)	GC (%)	N (ppm)
WT-NT	150	55024980	8253747000	96.86	92.33	48.61	29.17
WT-CN	150	40680972	6102145800	95.73	90.01	48.72	13.61
KO-NT	150	45106490	6765973500	96.79	92.25	48.12	29.55
KO-CN	150	49286176	7392926400	96.89	92.33	48.61	28.79

* WT-NT, untreated wild-type CD4$^+$ T cell (WT_UT); WT-CN, IL-25/IL-33 treated wild-type CD4$^+$ T cell (WT_IL-25/IL-33); KO-NT, untreated *Il17rb*$^{-/-}$ CD4$^+$ T cell (KO_UT); KO-CN, IL-25/IL-33 treated *Il17rb*$^{-/-}$ CD4$^+$ T cell (KO_IL-25/IL-33).

Supplementary Table S2. The summary of filtered data statistics of stimulated effector $CD4^+$ T cell isolated from lung of day 14 *C. neoformans* H99-infected wild-type and *Il17rb*$^{-/-}$ mice.

Sample name*	length	Clean reads	Clean bases	Q20 (%)	Q30 (%)	GC (%)	N (ppm)
WT-NT	146.58	54593162	8002022518	97.21	92.77	48.52	11.19
WT-CN	147.43	40402860	5956719848	96.11	90.45	48.7	6.06
KO-NT	146.36	44771526	6552981006	97.15	92.7	48.06	11.21
KO-CN	147.17	48976554	7208104760	97.2	92.71	48.58	11.18

---

* WT-NT, untreated wild-type $CD4^+$ T cell (WT_UT); WT-CN, IL-25/IL-33 treated wild-type $CD4^+$ T cell (WT_IL-25/IL-33); KO-NT, untreated *Il17rb*$^{-/-}$ $CD4^+$ T cell (KO_UT); KO-CN, IL-25/IL-33 treated *Il17rb*$^{-/-}$ $CD4^+$ T cell (KO_IL-25/IL-33).

Supplementary Table S5. Lists of significant unique KEGG pathways of up-regulated and down-regulated GO terms of stimulated effector CD4^{+} T cell isolated from the lung of day 14 *C. neoformans* H99-infected wild-type mice.

Pathway ID	Pathway	P-value	Q-value	Up-regulated		Down-regulated	
				Number	Gene name (logFC)	Number	Gene name (logFC)
ko00965	Betalain biosynthesis	0.001449	0.027926	1	*Ddc*(6.24492089)	0	
ko01230	Biosynthesis of amino acids	9.58E-05	0.00692	7	*Cth* (7.404238977), *Pycr1* (2.300267899), *Bcat1* (1.77561949), *Asns* (1.556580859), *Psat1* (1.457178474), Shmt2 (1.408234499), *Phgdh* (1.368801927)	1	*Arg2* (1.533019508)
ko00680	Methane metabolism	0.002772	0.040052	3	*Shmt2* (6.905818489), *Psat1* (6.811810501), *Phgdh* (4.862290375)	0	-
ko04512	ECM-receptor interaction	0.00256	0.038942	0	-	6	*Thbs2* (-5.997510106), *Lama1* (-5.830404978), *Itga9* (-2.345696913), *Col6a4* (-2.183637791), *Sdc1* (-1.756272246), *Cd36* (-1.485089866)
ko04640	Hematopoietic cell lineage	0.001281	0.027926	4	*Cd34* (6.054990746), *Cd59b* (6.054990746), *Csf2* (3.461837193), *Il5* (2.29817794)	3	*Cd33* (-3.212295806), *Cd36* (-1.485089866), *Ms4a1* (-1.436230508)
ko04380	Osteoclast differentiation	0.003742	0.045494	2	*Nox1* (4.237591211), *Ifng* (1.9963169)	6	*Fcgr4* (-5.830404978), *Fcgr3* (-2.903585336), *Btk* (-2.140364202), *Tnfrsf11a* (-1.843357203), *Ctsk* (-1.750036388), *Syk* (-1.727486686)
ko04060	Cytokine-cytokine receptor interaction	0.000406	0.011724	11	*Il9* (6.823528605), *Cd70* (3.477423716), *Csf2* (3.461837193), *Ccl24* (3.033495761), *Il13* (2.603474497), *Lif* (2.575368116), *Il5* (2.298177941), *Tnfrsf8* (2.171565757), *Cxcl10* (2.049305484), *Ifng* (1.9963169) *Il1rn* (1.41998184)	5	*Bmp8b* (-6.521060023), *Ccr1l1* (-5.830404978), *Lepr* (-3.353128266), *Tnfrsf11a* (-1.843357203), *Ccl6* (-1.680001136)
ko04610	Complement and coagulation cascades	0.00029	0.010478	4	*Cd59b* (6.054990746), *Serpine1* (2.481008435), *Serpind1* (1.980444799), *Masp2* (1.728530163)	4	*C1qb* (-6.626852741), *F13a1* (-6.626852741), *Cfb* (-5.830404978), *F7* (-1.934966688)
ko00270	Cysteine and methionine metabolism	0.001303	0.027926	5	*Cth* (7.404238977), *Srm* (1.896502321), *Bcat1* (1.77561949), *Psat1* (1.457178474), *Phgdh* (1.368801927)	0	-
ko05143	African trypanosomiasis	0.000124	0.007179	4	*Ido1* (2.900484424), *Vcam1* (2.366511366), *Ido2* (2.236773105), *Ifng* (1.9963169)	1	*Tlr9* (-4.140897601)
ko00330	Arginine and proline metabolism	0.000197	0.008137	5	*P4ha2* (2.953233208), *Azin2* (2.494326625), *Pycr1* (2.300267899), *Ckmt1* (2.06741969), *Srm* (1.896502321)	1	*Arg2* (1.533019508)
ko05310	Asthma	0.002068	0.037358	3	*Il9* (6.823528605), *Il13* (2.603474497), *Il5* (2.298177941)	0	-
ko00592	alpha-Linolenic acid metabolism	0.002402	0.038942	2	*Acaa1b* (2.131595961), *Pla2g4f* (1.960227593)	1	*Pla2g4b* (-2.150800529)
ko04724	Glutamatergic synapse	6.91E-05	0.00666	4	*Grm4* (6.24492089), *Pla2g4f* (1.960227593), *Gls2* (1.733878294), *Slc17a6* (1.49525301)	6	*Slc17a8* (-5.830404978), *Shank1* (-4.140897601), *Grin3b* (-3.532095621), S*lc1a2* (-3.25456612), *Gm4356* (-2.345013255), *Pla2g4b* (-2.150800529)
ko00901	Indole alkaloid biosynthesis	0	0	1	*Ddc*(6.24492089)	0	-
ko03320	PPAR signaling pathway	0.003332	0.045494	3	*Me1* (6.054990746), *Acaa1b* (2.131595961), *Fabp5* (1.60165836)	3	*Olr1* (-3.369019886), *Cd36* (-1.485089866), *Lpl* (-1.442353815)
ko00471	D-Glutamine and D-glutamate metabolism	0.001449	0.027926	1	*Gls2* (1.733878294)	0	-
ko04664	Fc epsilon RI signaling pathway	0.000149	0.007183	4	*Csf2* (3.461837193), *Il13* (2.603474497), *Il5* (2.298177941), *Pla2g4f* (1.960227593)	3	*Pla2g4b* (-2.150800529), Btk (-2.140364202), *Syk* (-1.727486686)
ko05144	Malaria	2.23E-05	0.003218	2	*Vcam1* (2.366511366), *Ifng* (1.9963169)	5	*Thbs2* (-5.997510106), *Tlr9* (-4.140897601), *Sdc1* (-1.756272246), *Lrp1* (-1.690593988), *Cd36* (-1.485089866)
ko00966	Glucosinolate biosynthesis	0.00049	0.012882	1	*Bcat1* (1.77561949)	0	-
ko00260	Glycine, serine and threonine metabolism	0.002489	0.038942	4	*Cth* (7.404238977), *Psat1* (1.457178474), *Shmt2* (1.408234499), *Phgdh* (1.368801927)	0	-
ko04950	Maturity onset diabetes of the young	0.003624	0.045494	1	*Slc2a2* (6.24492089)	2	*Pax6* (-3.965049559), *Bhlha15* (-2.156063829)
ko04657	IL-17 signaling pathway	0.003778	0.045494	6	*Csf2* (3.461837193), *Il13* (2.603474497), *Il5* (2.298177941), *Cxcl10* (2.049305484), *Ifng* (1.9963169), *Ptgs2* (1.984036828)	0	-
ko00460	Cyanoamino acid metabolism	0.000353	0.011341	2	*Ggt1* (6.938025674), *Shmt2* (1.408234499)	0	-

Supplementary Table S6. Lists of significant unique KEGG pathways of up-regulated and down-regulated GO terms of stimulated effector CD4+ T cell isolated from the lung of day 14 *C. neoformans* H99-infected *Il17rb*-/- mice.

Pathway ID	Pathway	P-value	Q-value	Up-regulated		Down-regulated	
				Number	Gene name (logFC)	Number	Gene name (logFC)
ko00983	Drug metabolism - other enzymes	0.001735	0.020819	5	*Upp2* (6.65716696), *Gstm1* (3.294862364), *Nme4* (2.403450395), *Gstt1* (2.092948019), *Tymp* (2.030637018)	1	*Mgst1* (2.740443464)
ko05321	Inflammatory bowel disease	0.000689	0.01737	2	*Il1b* (6.418755514), *Il22* (6.132991387)	3	*Il2* (-6.081500278), *Il23r* (-3.677517204), *H2-Ob* (-1.83969137)
ko05143	African trypanosomiasis	0.000363	0.010154	4	*Il1b* (6.418755514), *Ido1* (3.272458559), *Vcam1* (2.572104081), *Thop1* (1.588604529)	0	-
ko04014	Ras signaling pathway	0.001308	0.019395	6	*Gng7* (5.965633727), *Grin2b* (5.776282818), *Rapgef5* (3.002644197), *Pdgfa* (2.810007544), *Pdgfb* (2.513129965), *Fgf16* (2.513129965)	5	*Epha2* (-3.280355594), *Igf1* (-2.519970592), *Flt1* (-2.328504389), *Fgf13* (-1.839926933), *Pld1* (-1.727258768)
ko00920	Sulfur metabolism	0.000766	0.017555	0	-	2	*Papss2* (-2.935434685), *Tst* (-1.72422877)
ko05218	Melanoma	0.002608	0.029877	3	*Pdgfa* (2.810007544), *Pdgfb* (2.513129965), *Fgf16* (2.513129965)	2	*Igf1* (-2.519970592), *Fgf13* (-1.839926933)
ko04668	TNF signaling pathway	0.001395	0.019446	6	*Il1b* (6.418755514), *Il15* (6.132991387), *Cxcl10* (3.15463551), *Vcam1* (2.572104081), *Mmp9* (2.240237937), *Csf2* (2.237039428)	1	*Cxcl3* (2.037712207)
ko00966	Glucosinolate biosynthesis	0.0003	0.009447	1	*Bcat1* (1.408495543)	0	-
ko04657	IL-17 signaling pathway	0.000936	0.017778	4	*Il1b* (6.418755514), *Cxcl10* (3.15463551), *Mmp9* (2.240237937), *Csf2* (2.237039428)	2	*Cxcl3* (-2.037712207), *Il17re* (-1.668542018)
ko04672	Intestinal immune network for IgA production	0.00153	0.019446	1	*Il15* (6.132991387)	3	*Il2* (-6.081500278), *Ccr10* (-1.881294764), *H2-Ob* (-1.83969137)
ko04060	Cytokine-cytokine receptor interaction	1.13E-08	2.84E-06	13	*Cd70* (7.273643582), *Il1b* (6.418755514), *Il15* (6.132991387), *Il22* (6.132991387), *Tnfsfm13* (5.965633727), *Edar* (5.776282818), *Amh* (3.638296973), *Amhr2* (3.195341837), *Cxcl10* (3.15463551), *Csf2* (2.237039428), *Il1rn* (1.879948896), *Ccl3* (1.705750216), *Ccl1* (1.502159196)	8	*Gm44505* (-6.249056227), *Il2* (-6.081500278), *Prlr* (-5.891895418), *Acvrl1* (-4.408505045), *Il23r* (-3.677517204), *Cxcl3* (-2.037712207), *Ccr10* (-1.881294764), *Il17re* (-1.668542018)
ko04724	Glutamatergic synapse	0.001129	0.017778	4	*Dlgap1* (5.965633727), *Gng7* (5.965633727), *Grin2b* (5.776282818), *Grm4* (1.725299728)	3	*Grk3* (-6.773722804), *Trpc1* (-3.416304312), *Pld1* (-1.727258768)
ko00480	Glutathione metabolism	0.004334	0.042008	3	*Gpx7* (6.282937312) *Gstm1* (3.294862364), *Gstt1* (2.092948019)	1	*Mgst1* (2.740443464)
ko04540	Gap junction	0.001066	0.017778	3	*Pdgfa* (2.810007544), *Pdgfb* (2.513129965), *Tubb3* (2.361787347)	3	*Il31ra* (-5.891895418), *Gucy1b1* (-3.654917124), *Tuba8* (-2.7284729)
ko00240	Pyrimidine metabolism	9.24E-05	0.005822	6	*Upp2* (6.65716696), *Ak9* (5.776282818), *Entpd4* (5.776282818), *Nme4* (2.403450395), *Tymp* (2.030637018), *Dctd* (1.459697211)	0	-
ko05418	Fluid shear stress and atherosclerosis	0.000159	0.007198	7	*Il1b* (6.418755514), *Gstm1* (3.294862364), *Pdgfa* (2.810007544), *Vcam1* (2.572104081), *Pdgf*b (2.513129965), *Mmp9* (2.240237937), *Gstt1* (2.092948019)	3	*Thbd* (-6.535109992), *Mgst1* (-2.740443464), *Cav2* (-2.56907224)
ko04020	Calcium signaling pathway	0.003263	0.03426	5	*Nos2* (4.250647115), *Pdgfa* (2.810007544), *Pdgfb* (2.513129965), *Fgf16* (2.513129965), *P2rx3* (1.932247408)	5	*Htr5b* (-2.945135267), *P2rx5* (-2.482240785), *Flt1* (-2.328504389), *Pde1c* (-2.159221811), *Fgf13* (-1.839926933)
ko00910	Nitrogen metabolism	1.06E-05	0.001337	3	*Car14* (3.195341837), *Car8* (1.725299728), *Car13* (1.521490762)	1	*Car7* (-1.792668799)
ko05323	Rheumatoid arthritis	6.64E-05	0.005578	5	*Il1b* (6.418755514), *Il15* (6.132991387), *Csf2* (2.237039428), *Pth* (1.979341294), *Ccl3* (1.705750216)	3	*Flt1* (2.328504389), *Cxcl3* (-2.037712207), *H2-Ob* (-1.83969137)
ko04630	JAK-STAT signaling pathway	0.001102	0.017778	5	*Il15* (6.132991387), *Il22* (6.132991387), *Pdgfa* (2.810007544), *Pdgfb* (2.513129965), *Csf2* (2.237039428)	4	Gm44505 (-6.249056227), Il2 (-6.081500278), *Prlr* (-5.891895418), *Il23r* (-3.677517204)

Supplementary Table S6. Lists of significant unique KEGG pathways of up-regulated and down-regulated GO terms of stimulated effector $CD4^+$ T cell isolated from the lung of day 14 *C. neoformans* H99-infected *Il17rb*$^{-/-}$ mice. (Cont.)

Pathway ID	Pathway	P-value	Q-value	Up-regulated		Down-regulated	
				Number	Gene name (logFC)	Number	Gene name (logFC)
ko00261	Monobactam biosynthesis	0.0003	0.009447	0	-	1	*Papss2* (-2.935434685)
ko01051	Biosynthesis of ansamycins	0.000889	0.017778	0	-	1	*Tktl1* (-1.558895432)
ko00230	Purine metabolism	0.003716	0.037462	4	*Ak9* (5.776282818), *Entpd4* (5.776282818), *Ak1* (2.65052563), *Nme4* (2.403450395)	3	*Gucy1b1* (-3.654917124), *Papss2* (-2.935434685), *Pde1c* (-2.159221811)
ko00290	Valine, leucine and isoleucine biosynthesis	0.002898	0.031747	1	*Bcat1* (1.408495543)	0	-
ko05200	Pathways in cancer	0.004697	0.043835	12	*Smo* (7.593705725), *Il15* (6.132991387), *Gng7* (5.965633727), *Nos2* (4.250647115), *Wnt9a* (3.319466779), *Gstm1* (3.294862364), *Dll4* (3.059523636) *Pdgfa* (2.810007544), *Pdgfb* (2.513129965), *Fgf16* (2.513129965), *Mmp9* (2.240237937), *Gstt1* (2.092948019)	6	*Il2* (-6.081500278), *Il23r* (-3.677517204), *Mgst1* (-2.740443464), *Igf1* (-2.519970592), *Fgf13* (-1.839926933), *Pld1* (-1.727258768)
ko04061	Viral protein interaction with cytokine and cytokine receptor	0.001543	0.019446	3	*Cxcl10* (3.15463551), *Ccl3* (1.705750216), *Ccl1* (1.502159196)	3	*Il2* (-6.081500278), *Cxcl3* (-2.037712207), *Ccr10* (-1.881294764)
ko04015	Rap1 signaling pathway	0.000171	0.007198	6	*Grin2b* (5.776282818), *Rapgef5* (3.002644197), *Pdgfa* (2.810007544), *Pdgfb* (2.513129965), *Fgf16* (2.513129965), *Sipa1l2* (2.439340361)	6	*Rapgef4* (-3.357309687), *Epha2* (-3.280355594), *Igf1* (-2.519970592), *Flt1* (-2.328504389), *Fgf13* (-1.839926933), *Farp2* (-1.562216967)

Supplementary Table S7. Cryptococcal uptake of reference and clinical isolates of *C. neoformans*.

Groups*	Strains	Cryptococcal uptake (Cells/µL)	
		Mean	SD
Reference	H99	116.25	10.46
LU	CN008	33.75	3.54
	CN011	16.25	3.31
	CN014	30.42	7.11
HU	CN016	217.92	83.90
	CN018	245.00	76.88
	CN023	297.08	109.93

* *C. neoformans* clinical isolates with a low-uptake rate (LU) and high-uptake rate (HU) by macrophage.

Supplementary Table S8. Lung and brain fungal burdens of mice infected with clinical isolates of *C. neoformans* at 14 days postinfection.

Groups	Strains	LogCFU/g of Lung		LogCFU/g of Brain	
		Mean	SD	Mean	SD
LU	CN008	4.979529667	0.32690998	0.7685698	0.569026272
	CN011	6.360629667	0.373223044	0.4329158	0.194908469
	CN014	5.330121667	0.093114175	0.634801636	0.573959137
HU	CN016	5.332894667	0.083145652	3.028370333	0.195108245
	CN018	5.383461667	0.215369842	2.374330449	0.367631015
	CN023	5.167458	0.219900553	1.673112	0.058585899

---

[*] *C. neoformans* clinical isolates with a low-uptake rate (LU) and high-uptake rate (HU) by macrophage.
